# Regulation of Th2 responses by different cell types expressing the interleukin-31 receptor

**DOI:** 10.1186/s13223-017-0194-9

**Published:** 2017-04-17

**Authors:** Saburo Saito, Ayana Aoki, Iwao Arai, Shinya Takaishi, Haruyasu Ito, Nobutake Akiyama, Hiroshi Kiyonari

**Affiliations:** 1 Division of Molecular Immunology, Research Center for Medical Science, The Jikei University School of Medicine, 3-25-8 Nishi-shinbashi, Minato-ku, Tokyo, 105-8461 Japan; 2 Department of Dermatology, The Jikei University School of Medicine, 3-25-8 Nishi-shinbashi, Minato-ku, Tokyo, Japan; 3 Department of Otolaryngology, The Jikei University School of Medicine, 3-25-8 Nishi-shinbashi, Minato-ku, Tokyo, Japan; 4 Division of Rheumatology, Department of Internal Medicine, The Jikei University School of Medicine, 3-25-8 Nishi-shinbashi, Minato-ku, Tokyo, Japan; 5 Animal Resource Development Unit and Genetic Engineering Team, RIKEN Center for Life Science Technologies, 2-2-3 Minatojima-minamimachi, Chuou-ku, Kobe, Hyogo 650-0047 Japan

**Keywords:** IL-31 receptor, Th2, IgE, Cry j 2, Deficient mice

## Abstract

**Background:**

Interleukin-31 (IL-31) is a recently identified cytokine produced by Th2 cells that is involved in the development of atopic dermatitis-induced skin inflammation and pruritus. Its receptor, IL-31RA, is expressed by a number of cell types, including epithelial cells, eosinophils, and activated monocytes and macrophages. To date, however, the regulation of Th2 responses by distinct cell types and tissues expressing IL-31RA has not been well studied.

**Methods:**

In this study, Cry j 2, one of the major allergens of Japanese cedar pollen, was administered to IL-31RA-deficient or wild-type (WT) mice via nasal or intraperitoneal injection for induction of specific Th2 responses.

**Results:**

After nasal administration of Cry j 2, IL-31RA-deficient mice showed lower Cry j 2-specific CD4+ T cell proliferation, Th2 cytokine (IL-5 and IL-13) production, and Th2-mediated (IgE, IgG1, and IgG2b) antibody responses than WT mice. In contrast, IL-31RA-deficient mice administered Cry j 2 intraperitoneally showed stronger Th2 immune responses than WT mice.

**Conclusions:**

These results indicate that IL-31R signaling positively regulates Th2 responses induced by nasal administration of Cry j 2, but negatively regulates these responses when Cry j 2 is administered intraperitoneally. Collectively, these data indicate that the induction of antigen-specific Th2 immune responses might depend on tissue-specific cell types expressing IL-31RA.

## Background

Interleukin-31 (IL-31) is a recently identified cytokine produced by activated CD4+ Th2 cells that plays an important role in human T cell-mediated skin disease [1]. The expression of IL-31 has been shown to be correlated with the expression of the Th2 cytokines IL-4 and IL-13 in human skin diseases [2], and serum IL-31 has been shown to be higher in patients with atopic dermatitis [3]. When overexpressed in transgenic mice, IL-31 induces severe pruritus, which resembles eczema in humans [2]. Investigation of IL-31 transgenic mice has shown that overexpression of IL-31 results in the development of atopic dermatitis (AD)-like lesions, and that IL-31 expression is highly associated with Th2-skewed diseases [4]. These results indicate that IL-31 plays a role in the development and exacerbation of the Th2-associated disease AD.

In contrast to observations indicating that IL-31 is actively involved in the promotion of Th2-type diseases, others suggest that IL-31–IL-31R signaling negatively regulates Th2-type immune responses in the lungs following Schistosoma mansoni egg-induced inflammation [5]. In this study, the authors showed that IL-31RA-deficient mice developed exacerbated S. mansoni egg-induced Th2-type immune responses in the lungs and that loss of IL-31RA signaling resulted in enhanced antigen presentation by macrophages and increased Th2 cytokine expression by CD4+ T cells [5]. Furthermore, they showed that in response to Trichuris infection, IL-31RA-deficient mice exhibited increased Th2 cytokine responses in the mesenteric lymph nodes and elevated serum IgE and IgG1 levels as compared to WT mice [6]. In contrast, Bilsborough et al. reported that the susceptibility of IL-31RA KO mice [7] to exacerbated Th2-type diseases was an indirect result of IL-31RA deletion that causes an increased responsiveness to oncostatin M (OSM) and exacerbated production of OSM-inducible cytokines, such as IL-6, VEGF, and TIMP-1, during airway sensitization and challenge. These results indicate that the differential effects of IL-31 in distinct tissues may influence the distinct patterns of Th2-type immune responses, with, for example, positive effects in the skin, but negative effects in the lung and intestine.

On the basis of these previous findings, we assumed that Th2 immune responses are specifically regulated by different types of cells or tissues expressing the IL-31 receptor. To examine whether the reported exacerbated Th2-type response in IL-31RA KO mice [5, 6] has tissue-specific mechanisms, we investigated the antigen-specific Th2 responses in IL-31RA-deficient mice administered an allergen nasally or intraperitoneally.

## Methods

### Mice

**Fig. 1:**
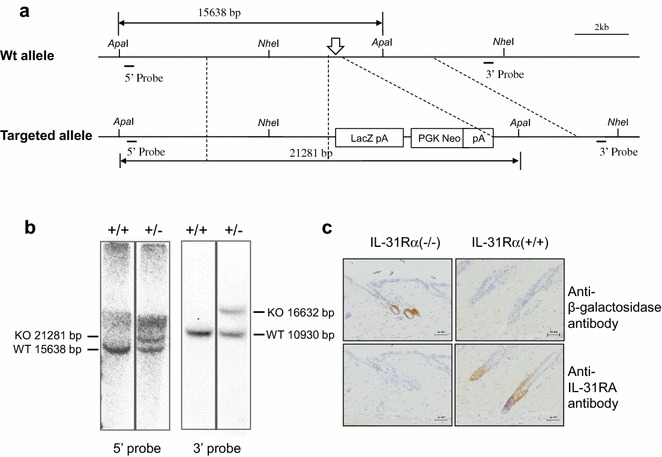
Generation of IL-31RA-deficient mice. To generate C57BL/6-IL-31RA^tLacZ/+^ knock-out (KO) mice, we used homologous recombination in embryonic stem (ES) cells to create a mutant allele in which exon 4 of the IL-31RA gene was replaced by a cassette expressing the selective marker neomycin transferase (**a**). Two recombinant ES cells were found to be IL-31RA^tm1(LacZ)^. Chimeric mice were mated with C57BL/6 mice to produce mutant IL-31RA^+/LacZ(+/−)^ progeny. The generation of mutant IL-31RA^+/−^ mice was verified by Southern blot analysis (**b**). The IL-31RA^+/−^ allele was then backcrossed to C57BL/6 mice for 15 more generations using male IL-31RA^+/−^. Homozygous IL-31RA^−/−^ and WT (IL-31RA^+/+^) littermates were generated by intercrossing IL-31RA^+/−^ mice from the 15th generations of backcrossed mice. Heterozygous IL-31RA^+/−^ and homozygous IL-31RA^−/−^ littermates were generated by crossing IL-31RA^+/−^ mice with homozygous IL-31RA^−/−^ mice. The expression of IL-31RA or beta-galactosidase of skin from IL-31RA^+/+^ and IL-31RA^−/−^ mice was revealed by immunohistological staining with antibodies against each antigen (**c**)

C57BL/6 mice were purchased from Sankyo Laboratories (Tokyo, Japan) and were housed in our facilities under specific pathogen-free conditions. All experiments were performed following the Animal Experimentation Guidelines of The Jikei University School of Medicine and the RIKEN Kobe Branch. The IL-31RA mutant (Accession No. CDB1012K: http://www2.clst.riken.jp/arg/mutant%20mice%20list.html) was established as follows. To generate IL-31RA deficient mice, homologous recombination in embryonic stem (ES) cells was used to create a mutant allele in which exon 4 of the IL-31RA gene was replaced with a cassette expressing the selective marker neomycin transferase. In brief, homologous regions (5′5 8001 bp, 3′: 3074 bp) were subcloned into a knock-in vector (DT-A-pA/lox71/LacZ-pA/frt/PGK-Neo/frt/loxP/pA: http://www2.clst.riken.jp/arg/cassette.html) and electroporated into mouse ES cells. Two recombinant ES cells were found to be IL-31RA^tm1(LacZ)^. Homologously integrated ES cells were injected into 8-cell-stage zygotes (Fig. 1a). Chimeric mice were mated with C57BL/6 mice to produce mutant IL-31RA^+/LacZ(+/−)^ progeny. The generation of mutant IL-31RA^+/−^ mice was verified by Southern blot analysis (Fig. 1b). Digestion with ApaI followed by hybridization of membranes with probe 1 (a 900-bp genomic DNA fragment obtained by PCR: GenBank AC154767.2, nt 49651–50550) yielded a 15,638-bp fragment for the WT allele and a 21,281-bp fragment for the correctly targeted mutant allele. Similarly, digestion of genomic DNA with NheI followed by hybridization of membranes with probe 2 (a 800-bp genomic DNA fragment obtained by PCR: GenBank NC_000079.6, nt 112555274–112556073) yielded a 16,632-bp fragment for the WT allele and a 10,930-bp fragment for the correctly targeted mutant allele. To reduce heterozygosity, the IL-31RA^+/−^ allele was then backcrossed to C57BL/6 mice for 15 more generations using IL-31RA^+/−^ males. Disruption of IL-31RA was identified by PCR using the corresponding primers to give a 602-bp product (primer1: 5′-caaagcgccattcgccattcaggctgcgca-3′,primer2: 5′-tgtgcattgtgagtgggtgagtggtatgca-3′). The presence of wild-type IL-31RA was identified as a 377-bp product (primer 2 and primer 3: 5′-tgaatttgcagaggaaagagaatgcccaca-3′).

Homozygous IL-31RA^−/−^ and WT (IL-31RA^+/+^) littermates were generated by intercrossing IL-31RA^+/−^ mice from the 15th generation of backcrossed mice. Heterozygous IL-31RA^+/−^ and homozygous IL-31RA^−/−^ littermates were generated by crossing IL-31RA^+/−^ mice with homozygous IL-31RA^−/−^ mice.

### Immunohistochemistry for IL-31RA and beta-galactosidase

To examine the expression of IL-31RA or beta-galactosidase (β-Gal) from WT C57BL/6 mice (IL-31RA^+/+^) and IL-31RA-deficient (IL-31RA^−/−^) mice, skin specimens were fixed for 3 days in 10% normal buffered formalin and embedded in paraffin using standard techniques. Five-micrometer sections were heated at 60 °C for 30 min for tissue adhesion. Slides were subsequently dewaxed by incubation in xylene and then rehydrated in 100, 95, and 70% EtOH. Finally, the slides were rinsed with Tris-buffered saline Tween buffer, and prepared as recommended by the manufacturer. Endogenous peroxidase was blocked with 4.5% H_2_O_2_ in MeOH for 30 min at room temperature. A protein block (phosphate-buffered saline block containing 10% normal goat serum) was applied overnight at 4 °C.

To examine the expression of IL-31RA, we prepared rabbit anti-mouse IL-31RA polyclonal antibodies as follows. A partial peptide of murine IL-31RA, mGPL 65–86 (YSDNATEASYSFPRSCAMPPDI), was synthesized by referring to [8] and conjugated to keyhole limpet hemocyanin. Rabbits were immunized with the conjugates and Freund’s Complete Adjuvant. After booster shots, whole blood from immunized rabbits was collected and serum was separated. Serum antibodies that recognize IL-31RA were purified using a column containing Sepharose-bound mGPL 65–86 peptide and the antigen-binding activity of the purified antibodies to the peptide was checked by ELISA. To examine the expression of β-Gal, an anti-beta β-Gal antibody (Abcam PLC, Cambridge, US) was used as a primary antibody and biotin-conjugated goat anti-chicken IgY (H+L) polyclonal antibody (GeneTex Inc, CA, USA) was used as a secondary antibody for β-Gal staining.

### Immunization

To induce allergen-specific Th2 responses, mice were administered an allergen nasally or intraperitoneally. For nasal administration, 2.5 µg of Cry j 2 (Hayashibara Biochemical Laboratories, Okayama, Japan), one of the major allergens of Japanese cedar pollen, was dissolved in 4 μl of PBS, and simply administered intranasally 15 times (5 times per week). For intraperitoneal immunization, mice were intraperitoneally injected with 20 µg of Cry j 2 in 0.2 ml of PBS without any adjuvants once a week for 5 weeks.

### T-cell proliferation assay

Seven days after the last immunization, the spleen and lymph node cells were collected. Cry j 2-specific T-cell proliferative responses were determined by an in vitro [^3^H] thymidine incorporation assay. RPMI-1640 medium (Thermo Fisher Scientific Inc., MA, USA) supplemented with 1% normal mouse serum was used to suspend cells and 8 × 10^5^ cells were seeded into each well of 96-well plates (Nunc Microwell 96F; Thermo Fisher Scientific Inc.) and cultured with 2.5 µg/ml of Cry j 2 for 88 h. The cultures were pulsed with 0.5 µCi of [^3^H] thymidine (American Radiolabeled Chemicals Inc., USA) for the last 16 h in Nunc Maxisorp plates (Kamnstrup, Denmark). The cells were harvested using a Micro 96 Harvester (Skatron Instruments, Norway), and [^3^H] thymidine uptake by the cells was determined by measuring the radioactivity using a liquid scintillation counter (LSC-6000; ALOKA, Tokyo, Japan). T-cell proliferation was expressed as a stimulation index (SI), which represents the ratio of [^3^H] thymidine incorporation in cultures with and without antigen. Responses with SIs greater than 2.0 were considered positive.

### Cytokine assay

Spleen or lymph node cells were stimulated with 2.5 µg/ml of Cry j 2 and supernatants were collected at 72 h and stored at −20 °C for cytokine detection assays. The concentrations of IL-5, IL-13, and IFN-γ were determined using standard sandwich ELISA protocols (IL-5: BD Biosciences, NJ, USA; IL-13: Thermo Fisher Scientific Inc.; IFN-γ: BD Biosciences).

### ELISA

For the assay of Cry j 2-specific IgE, IgG1, IgG2a, and IgG2b, we used serum collected from mice 7 days after immunization. Antigen-specific IgE antibody titers were measured by IgE capture ELISA as previously described [9], with several modifications. Microtiter plates were incubated with anti-mouse IgE (BD Biosciences, CA, USA) capture antibody overnight at 4 °C, washed, and blocked with PBS containing 10% FCS for 1 h. The plates were incubated with serially diluted serum samples for 2 h. After washing, 0.3 µg/ml of biotinylated Cry j 2 was added to each well for further incubation for 1 h at room temperature. The plates were washed and streptavidin-horseradish peroxidase (HRP) conjugates (BD Biosciences) was added and incubated for 30 min. After 5 times washing, tetramethylbenzidine (TMB) substrate solution was added to each well and incubated for 30 min in the dark, and 2 N H_2_SO_4_ solution was added to each well to stop the reactions. The absorbance in each well was measured with a microplate reader (Bio-Rad, CA, USA). To calculate the anti-Cry j 2 IgE antibody titer, sera of five C57 BL/6 mice immunized five times with Cry j 2 in the peritoneal cavity were pooled and a standard curve was prepared to calculate anti-Cry j 2 IgE antibody by measuring total IgE. There was a correlation between total IgE and the Cry j 2-specific IgE antibody titer in the diluted samples from pooled sera. On the other hand, anti-Cry j 2 IgE antibody could not be detected in non-immunized mice (data not shown).

Antigen-specific IgG, IgG1, IgG2a, and IgG2b antibody titers were measured by indirect ELISA. Microplates, which were coated with Cry j 2 (1 µg/mL), were blocked with FCS, and serum samples were added to the wells and incubated for 2 h at room temperature. After the plates were washed, HRP-conjugated isotype-specific antibody was added to the wells. The bound antibodies were detected by adding TMB substrate solution, and color development was assessed by measuring the absorbance at 450 nm.

### Statistical analysis

Means were compared using the unpaired or paired *t* test in GraphPad Prism version 6.02 (GraphPad Software Inc., San Diego, CA, USA). P values of <0.05 were regarded significant.

## Results

### Generation of IL-31RA-deficient mice

To investigate the function of endogenous IL-31–IL-31R interactions, IL-31RA-deficient mice were generated as IL-31RA knock-in mice by homologous recombination, as shown in Fig. 1a. Correct homologous recombination was confirmed by Southern blot analysis (Fig. 1b). The phenotypes were not significantly different in the two IL-31RA^+/LacZ(−)^ knock-in mouse lines. Inheritance of WT and mutant alleles was followed by PCR analysis of genomic DNA obtained from tail biopsies with pairs of primers specific for WT or mutant alleles. Digestion with ApaI followed by hybridization of membranes with a 5′ probe (900-bp) of a genomic DNA fragment obtained by PCR with oligos and a 3′ probe (800-bp) yielded a 15,638-bp fragment for the WT allele and a 21,281-bp fragment for the correctly targeted mutant allele. The IL-31RA^+/−^ allele was then backcrossed to C57BL/6 mice for 15 more generations using males IL-31RA^+/−^. Disruption of IL-31RA was identified by PCR with the corresponding primers.

Homozygous IL-31RA^−/−^ and WT (IL-31RA^+/+^) littermates were generated by intercrossing IL-31RA^+/−^ mice from the 15th generation of backcrossed mice. Heterozygous IL-31RA^+/−^ and homozygous IL-31RA^−/−^ littermates were generated by crossing IL-31RA^+/−^ mice with homozygous IL-31RA^−/−^ mice. As shown in Fig. 1c, the absence of IL-31RA expression in the skin of IL-31RA-deficient mice was confirmed by immunohistological staining with antibodies against IL-31RA or β-Gal. Staining with the anti-IL-31RA antibody revealed that IL-31RA was expressed in the hair roots of the skin of the WT mice but not in that of IL-31RA^−/−^ mice. In contrast, β-Gal was found to be expressed in the skin hair roots of the IL-31RA^−/−^ mice but not in that of WT mice.

### IL-31RA-deficient mice show decreased specific Th2 immune responses following nasal injection of Cry j 2

**Fig. 2:**
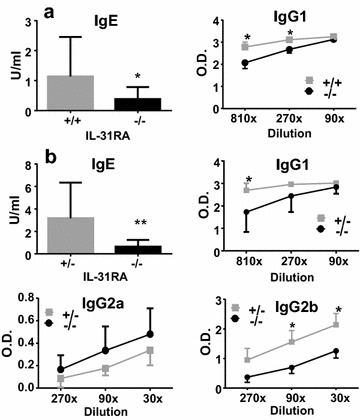
IL-31RA^−/−^ mice nasally injected with Cry j 2 exhibit decreased antigen-specific Th2-mediated antibody responses. Serum from WT (IL-31RA^+/+^, N = 6) and KO (IL-31RA^−/−^, N = 7) mice following 15 nasal administrations of Cry j 2 was assayed for antigen-specific IgE and IgG1 antibodies (**a**). Littermate IL-31RA^−/−^ mice (N = 15) and IL-31RA^+/−^ control mice (N = 10) were nasally administered Cry j 2 antigen five times a week for induction of specific Th2 responses. Serum from littermate mice following 15 nasal administrations was assayed for antigen-specific IgE, IgG1, IgG2a, and IgG2b levels (**b**) by ELISA. The data shown are of one experiment representative of three independent experiments. Data are presented as the mean ± SD. Unpaired *t* test; *P < 0.05. **P < 0.01

**Fig. 3:**
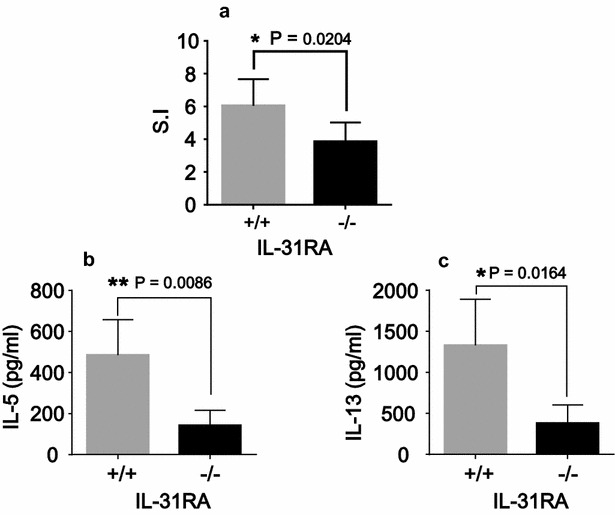
IL-31RA^−/−^ mice nasally injected with Cry j 2 show decreased antigen-specific proliferative and Th2 cytokine responses. 7 days after 15 nasal administrations of Cry j 2, spleen cells from WT (IL-31RA^+/+^, N = 6) and KO (IL-31RA^−/−^, N = 7) mice were cultured for 88 h in the presence or absence of Cry j 2 (0.1 μg/ml) and pulsed for the last 16 h with 0.5 μCi of tritiated thymidine. The results of T-cell proliferation (**a**) are expressed in terms of a stimulation index (SI) calculated by dividing counts per minute (cpm) in the presence of antigen with cpm in the absence of antigen. Supernatants obtained from a 72-h cell culture were assayed by ELISA for IL-13 (**b**) and IL-5 (**c**). Data are presented as the mean ± SD

In order to clearly analyze the differential effects of tissue-specific IL-31R signaling on Th2 immune responses, mice were injected nasally or intraperitoneally with the Th2 antigen Cry j 2. First, to examine the positive role of IL-31–IL-31R interactions in the skin on Th2 immune responses, IL-31RA-deficient and WT mice were injected nasally with Cry j 2, one of the major allergens of Japanese cedar pollen, which induces Th2 polarized and allergic inflammation [10]. Following repeated nasal administration of Cry j 2, the Cry j 2-specific serum IgE and IgG1 levels in IL-31RA-deficient mice were significantly lower than those observed in the serum of WT mice injected nasally with Cry j 2 (Fig. 2a). To evaluate the effects of tissue-specific IL-31R signaling on Th2 immune responses more accurately, we used the Cry j 2 allergen as an immunogen without any adjuvants to induce Th2 immune responses, as Cry j 2 has protease activity similar to that of Th2 adjuvants [11]. However, when Cry j 2 was administered in a mixture with Alum, there was no difference in the level of induced Th2 immune response between IL-31RA-deficient and WT mice (data not shown), even though Alum adjuvants strongly induce antigen-specific Th2 immune responses [12]. To confirm our previous observations that IL-31–IL-31R interactions positively influence Th2 immune responses following nasal administration of Cry j 2 (Fig. 2a), heterozygous IL-31RA^+/−^ and homozygous IL-31RA^−/−^ littermates were generated by crossing IL-31RA^+/−^ with IL-31RA^−/−^ mice. The littermates were nasally administered Cry j 2 and examined for Cry j 2-specific Th2 immune responses. Consistent with previous findings, in IL-31RA^−/−^, Cry j 2-specific IgE and IgG1 (Fig. 2b) serum levels were significantly lower than those in IL-31RA^+/−^ mice. In addition, serum levels of IgG2b (Fig. 2b), which are influenced by Th2 immune responses [13], were also significantly lower in IL-31RA^−/−^ mice than in IL-31RA^+/−^ mice. In contrast, serum IgG2a (Fig. 2b), which is influenced by IFN-γ secreted by Th1-cells [14], did not differ between the two strains. Furthermore, in IL-31RA-deficient mice, not only antigen-specific proliferative responses (Fig. 3a) of spleen cells but also secretion levels of the Th2 cytokines IL-5 (Fig. 3b) and IL-13 (Fig. 3c) were significantly lower than those in WT mice injected nasally with Cry j 2. These results demonstrated that IL-31R signaling following nasal administration of an allergen can positively regulate the Th2 immune response, which is consistent with the findings of numerous previous studies that have shown that IL-31R signaling exacerbates Th2-associated immune responses [1–3, 15–17].

**Fig. 4:**
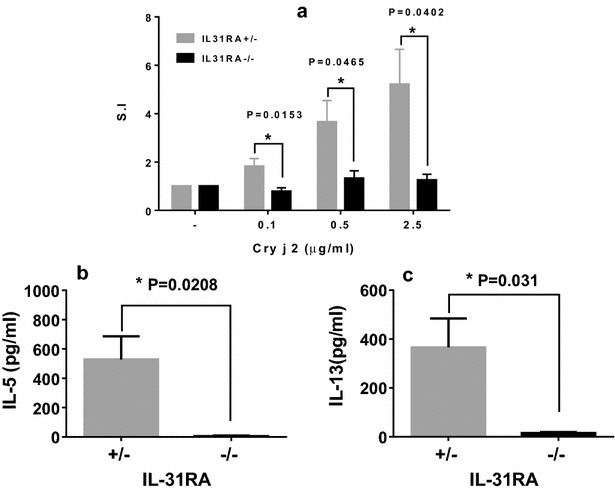
Decreased antigen-specific proliferative and Th2 cytokine responses of spleen cells from IL-31RA^−/−^ mice nasally injected with Cry j 2. Seven days after 19 nasal administrations of Cry j 2, spleen cells from six representative IL-31RA^−/−^ and littermate control IL-31RA^+/−^ mice were cultured for 88 h in the presence or absence of the indicated dose of Cry j 2 and pulsed for the last 16 h with 0.5 μCi of tritiated thymidine to analyze antigen-specific T-cell proliferative responses (**a**). Supernatants from 72-h cell cultures were assayed for IL-13 (**b**) and IL-5 (**c**). Data are presented as the mean ± SD

**Fig. 5:**
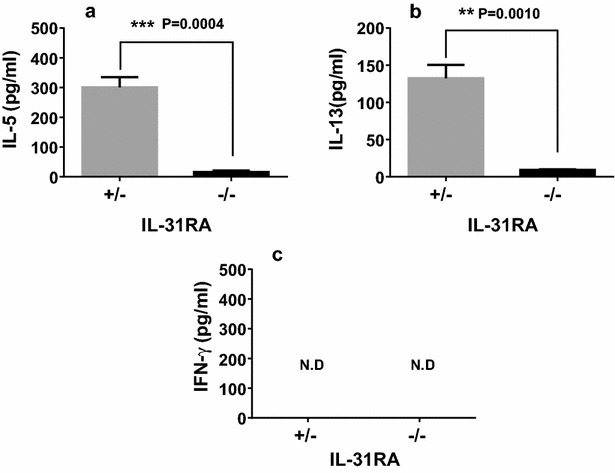
Decreased antigen-specific Th2 cytokine responses of submandibular lymph node cells from IL-31RA^−/−^ mice nasally injected with Cry j 2. 7 days after 19 nasal administrations of Cry j 2, submandibular lymph node cells from six representative IL-31RA^−/−^ and littermate control IL-31RA^+/−^ mice were pooled and cultured. Supernatants from a 72-h cell culture were assayed by ELISA for IL-5 (**a**), IL-13 (**b**), and IFN-γ (**c**). Data are presented as the mean ± SD

Furthermore, spleen cells isolated from IL-31RA^−/−^ mice nasally administered Cry j 2 showed significantly lower levels of Cry j 2-specific T-cell proliferative responses (Fig. 4a) and Th2 cytokine IL-5 and IL-13 secretion than did IL31 RA^+/−^ mice nasally administered Cry j 2 (Fig. 4b, c). In addition, IL31 RA^−/−^ mice nasally administered Cry j 2 in which submandibular lymph node cells were draining showed significantly lower levels of Cry j 2-specific Th2 cytokine IL-5 and IL-13 secretion than IL31 RA^+/−^ mice nasally administered Cry j 2 (Fig. 5a, b), whereas IFN-γ secretion was not detected in either strain of immunized mice (Fig. 5c). Collectively, these results strongly demonstrated that IL-31R signaling following nasal administration of an allergen can positively regulate Th2-associated immune responses.

### IL-31RA-deficient mice intraperitoneally injected with Cry j 2 show heightened specific Th2 immune responses

**Fig. 6:**
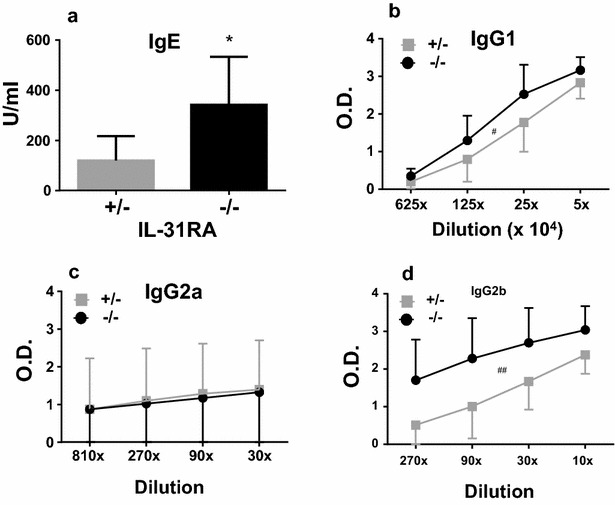
IL-31RA^−/−^ mice administered intraperitoneally with Cry j 2 exhibit enhanced antigen-specific Th2-mediated antibody responses. Littermate IL-31RA^−/−^ and IL-31RA^+/−^ control mice were intraperitoneally administered once a week with Cry j 2 for induction of antigen-specific Th2 responses. 7 days after three intraperitoneal administrations of Cry j 2, serum from IL-31RA^−/−^ (N = 5) and littermate control IL-31RA^+/−^ (N = 5) mice were collected and assayed for Cry j 2-specific IgE (**a**), IgG1 (**b**), IgG2a (**c**), and IgG2b (**d**) antibody responses. Data are presented as the mean ± SD. Unpaired *t* test; *P < 0.05. Paired *t* test; ^#^P < 0.05, ^##^P < 0.01

**Fig. 7:**
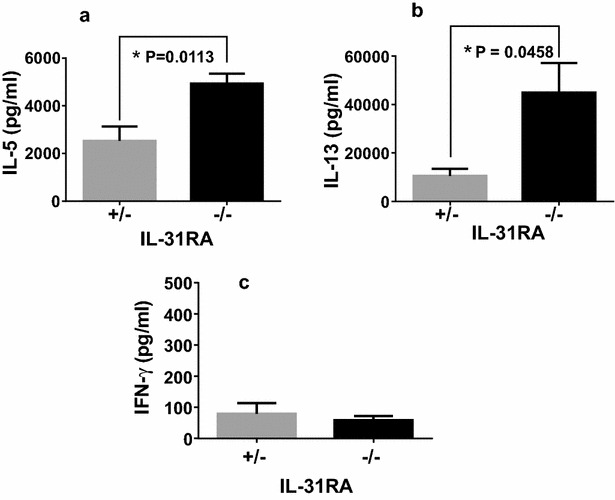
IL-31RA^−/−^ mice intraperitoneally administered Cry j 2 show enhanced antigen-specific proliferative and Th2 cytokine responses. For the analysis of Th2 cytokine responses, spleen cells were collected from mice 7 days after five intraperitoneal administrations of Cry j 2 and cultured with Cry j 2. Supernatants from 72-h cell cultures were assayed for IL-5 (**a**), IL-13 (**b**), and IFN-γ (**c**). The data shown are of one experiment representative of five independent experiments with 4 to 10 mice per group. Data are presented as the mean ± SD

To investigate whether IL-31R signaling in different cells and tissues influences the patterns of Th2 immune responses, IL-31RA-deficient (IL-31RA^−/−^) and IL-31RA^+/−^ littermate mice were intraperitoneally administered Cry j 2. Following a repeated cycle of intraperitoneal administration with Cry j 2, the level of Cry j 2-specific serum IgE (Fig. 6a), IgG1 (Fig. 6b), and IgG2a (Fig. 6c) in IL-31RA-deficient mice were significantly higher than IL-31RA^+/−^ mice intraperitoneally administered Cry j 2. On the other hand, the serum level of IgG2a (Fig. 6c) did not differ between the two strains. Furthermore, in IL-31RA-deficient mice, the secretion levels of Th2 cytokines IL-5 and IL-13 (Fig. 7a, b) were significantly higher than those in IL-31RA^+/−^ mice injected intraperitoneally with Cry j 2. In contrast, IFN-γ production by Th1-cells did not differ between the two strains. These data are consistent with the increase in Th2 cytokines observed in the draining lymph node of the lungs in IL-31RA^−/−^ mice following S. mansoni egg injection [5], and support a role for IL-31–IL-31R interactions in limiting intestinal Th2 cytokine production following Trichuris infection [6]. These results demonstrate that IL-31R signaling in mice intraperitoneally administered Cry j 2 negatively regulates the Th2-associated immune responses, even though IL-31R signaling following nasal administration positively regulates the Th2 responses. Collectively, these data demonstrated that IL-31R signaling following nasal administration of an allergen positively regulates, whereas that following intraperitoneal administration negatively regulates, antigen-specific Th2 responses. These results indicated that regulation of the Th2 immune response depends on the cell types expressing IL-31RA, in which IL-31R signaling plays an important role.

## Discussion

In this study, in order to investigate the differential effects of cell- and tissue-specific IL-31R signaling on the induction of antigen-specific Th2 immune responses, Cry j 2, one of the major allergens of Japanese cedar pollen, was administered either nasally or intraperitoneally into IL-31RA-deficient and WT mice. After repeated nasal administration of Cry j 2, IL-31RA-deficient mice showed significantly lower levels of Cry j 2-specific Th2 proliferative responses, IL-5 and IL-13 cytokine production, and Th2-mediated Cry j 2-specific IgE, IgG1, and IgG2b antibody responses than IL-31RA^+/+^ WT mice. Even when compared with IL-31RA^+/−^ littermate mice, IL-31RA-deficient mice nasally administered Cry j 2 showed lower levels of Th2 immune responses. In contrast, IL-31RA-deficient mice intraperitoneally administered Cry j 2 showed significantly higher levels of Cry j 2-specific Th2 proliferative responses, IL-5 and IL-13 cytokine production, and Th2-mediated Cry j 2-specific IgE, IgG1, and IgG2b antibody responses than did IL-31RA^+/−^ littermates. In contrast, even if IL-31RA was deficient, Th1 cytokine (IFN-γ) production and Th1-mediated IgG2a antibody response were not affected. These data indicate that IL-31–IL-31R interactions in mice receiving nasal administration of an allergen positively regulate Th2 immune responses, whereas in those receiving intraperitoneal administration, these interactions negatively regulate Th2 immune responses.

Our finding that IL-31R signaling following nasal administration of an allergen positively regulates the Th2 immune responses may be explained by the findings of previous studies from the perspective of the cell type expressing IL-31RA involved in induction of the Th2 immune response [18, 19]. These studies showed that IL-31 induces cytokine and chemokine production from human bronchial epithelial cells through activation of mitogen-activated protein kinase signaling pathways related to an allergic response. Stott et al. [19] also indicated primary bronchial epithelial cells treated with IL-31 induced proinflammatory genes, such as CCL2 and granulocyte colony-stimulating factor, suggesting the proinflammatory role of IL-31 in amplifying existing Th2 reactions.

In contrast to our observation that IL-31R signaling following nasal administration of an allergen positively regulates Th2 immune responses, we found that intraperitoneal administration of Cry j 2 resulted in negative regulation of antigen-specific Th2 immune responses. Previous reports have also indicated that IL-31 negatively regulates the development of the Th2-type response [5, 6], which is in accordance with our findings regarding intraperitoneal administration. Perrigoue et al. showed that IL-31RA-deficient mice exhibited elevated expression of Th2 cytokines and developed exacerbated airway inflammation following exposure to S. mansoni eggs, indicating a negative regulatory role for endogenous IL-31–IL-31R interactions in limiting type 2 inflammation in the lungs [5]. Furthermore, they showed that in the absence of IL-31RA expression, the magnitude of helminth-induced type 2 immune responses is significantly increased, as illustrated by heightened Th2 cytokine production, elevated IgE and IgG1 antibodies, and enhanced goblet cell responses in IL-31RA^−/−^ mice, culminating in accelerated worm expulsion [6]. These results are consistent with our findings that IL-31RA-deficient mice administered Cry j 2 intraperitoneally only showed significantly higher levels of Cry j 2-specific Th2 proliferative responses, IL-5 and IL-13 cytokine production, and serum IgE and IgG1 antibodies than WT mice. From the viewpoint of cell type-specific IL-31R signaling, Perrigoue et al. revealed that T cells co-cultured with IL-31RA^−/−^ macrophages proliferated to a greater extent than those cultured with WT macrophages under both neutral and Th2-polarizing conditions [5]. These data suggest that IL-31R signaling of macrophages negatively regulates the T cell stimulatory function. Collectively, our findings lead us to propose the idea that intraperitoneal administration of an allergen negatively regulates the Th2 immune response through IL-31R signaling of abdominal macrophages. Bilsborough et al. [7] indicated that the absence of IL-31RA expression in mice results not only in increased responsiveness to OSM but also in increased basal levels of VEGF in the lungs of naive animals, which may promote their susceptibility to Th2 sensitization [20], although levels of cytokines in the BALF of antigen-sensitized mice after treatment with anti-OSM monoclonal antibodies were not inhibited. Although the mechanisms of cell- or tissue-specific IL-31R signaling for regulating Th2 immune cells remain to be elucidated, determining the tissue distribution of receptor variants of IL-31RA and OSMR [1] may be critical to our understanding of the regulation of Th2 immune responses induced by different routes of administration.

## Conclusions

The results of the present study indicate that IL-31R signaling positively regulates Th2 responses induced by nasal administration of an allergen but negatively regulates these responses following intraperitoneal administration. Collectively, the data suggest that regulation of Th2 immune responses might be dependent on tissue-specific cell types expressing IL-31RA.

## Abbreviations

IL-31: interleukin-31
OSMR: oncostatin M receptor
KO: knock-out
IL-31R: IL-31 receptor
Cry j 2: cryptomeria japonica 2
